# DNA methylation signatures provide novel diagnostic biomarkers and predict responses of immune therapy for breast cancer

**DOI:** 10.3389/fgene.2024.1403907

**Published:** 2024-06-06

**Authors:** Zhishan Chen, Han Jiang, Qingqing Qin, Qiyuan Li, Liqing Hong

**Affiliations:** ^1^ Department of Breast and Thyroid Surgery, Nan’an Hospital, Quanzhou, China; ^2^ Department of General Surgery, The First Affiliated Hospital of Xiamen University, School of Medicine, Xiamen University, Xiamen, China; ^3^ School of Medicine, Xiamen University, Xiamen, China

**Keywords:** DNA methylation, breast cancer, prognosis, signature, immune therapy

## Abstract

Breast cancer (BRCA) is one of the most common malignant tumors affecting women worldwide. DNA methylation modifications can influence oncogenic pathways and provide potential diagnostic and therapeutic targets for precision oncology. In this study, we used non-parametric permutation tests to identify differentially methylated positions (DMPs) between paired tumor and normal BRCA tissue samples from the Cancer Genome Atlas (TCGA) database. Then, we applied non-negative matrix factorization (NMF) to the DMPs to derive eight distinct DNA methylation signatures. Among them, signatures Hyper-S3 and Hypo-S4 signatures were associated with later tumor stages, while Hyper-S1 and Hypo-S3 exhibited higher methylation levels in earlier stages. Signature Hyper-S3 displayed an effect on overall survival. We further validated the four stage-associated signatures using an independent BRCA DNA methylation dataset from peripheral blood samples. Results demonstrated that 24 commonly hypomethylated sites in Hypo-S4 showed lower methylation in BRCA patients compared to healthy individuals, suggesting its potential as an early diagnostic biomarker. Furthermore, we found that methylation of 23 probes from four stage-related signatures exhibited predictive power for immune therapy response. Notably, methylation levels of all three probes from the Hypo-S4 and activity of the Hypo-S4 demonstrated highly positive relevance to PD-L1 gene expression, implying their significant predictive values for immunotherapy outcomes. GO and KEGG pathway enrichment analysis revealed that genes with these 23 immunotherapy-related methylation probes are mainly involved in glycan degradation or protein deglycosylation. These methylation signatures and probes may serve as novel epigenetic biomarkers for predicting tumor immunotherapy response. Our findings provide new insights into precision oncology approaches for BRCA.

## 1 Introduction

Breast cancer (BRCA) is one of the most common malignant tumors in women worldwide and a major cause of cancer-related deaths among women globally (Sung et al., 2021). The number of new BRCA cases is on the rise annually across the world, particularly in developing countries. Studies have shown that accounts for approximately 60%–90% of BRCA-related deaths are attributed to metastasis of tumor (Dillekas et al., 2019; Krishnan et al., 2021). Thus, early detection is critical for BLCA treatment and prognosis. Mammography and ultrasound have been utilized for standardizing breast lesion risk assessment, among which mammography screening reduced breast cancer mortality by ∼20% (Screening, 2012; Christiansen et al., 2022). However, they are susceptible to high false positive rates, resulting in unnecessary biopsies. Particularly in case of high-density breast tissue, the detection sensitivity is compromised. Therefore, there is a need for new precise detection methods to compensate for the deficiency in breast lesion detection.

The occurrence and progression of tumors are accompanied by a series of reconstruction processes of the genome and epigenome (Chakravarthi et al., 2016; Ushijima et al., 2021). Among them, DNA methylation is an epigenetic mechanism that regulates gene expression and chromatin structure in a complex way affecting gene expression. Studies have confirmed that abnormal methylation patterns play an important role in the occurrence and progression of breast cancer and other malignant tumors (Kulis and Esteller, 2010; Ma et al., 2023). Since the DNA methylation modification process precedes protein translation, abnormal methylation patterns can be detected in the early stages of cancer development, and thus DNA methylation markers may have greater value in early diagnosis of breast cancer compared to detecting cancer-related protein expression levels. Currently, the most widely used diagnostic application related to methylation modification is the Sept9 methylation detection for early diagnosis of colorectal cancer based on cell-free DNA (cf-DNA) in peripheral blood (Galanopoulos et al., 2017; Fu et al., 2018), but its accuracy for early diagnosis is less than 80%. For detecting breast cancer, CA153 antigen detection is used as a breast cancer diagnostic method (Stefan-van Staden and van Staden, 2013; Tang et al., 2016). The antigen detected by this assay kit is significantly elevated in the serum of late-stage breast cancer patients. Its effectiveness for early breast cancer diagnosis is also not ideal. Therefore, it is more commonly applied for preoperative detection and monitoring disease progression after surgery. Developing new methods for early breast cancer diagnosis, especially DNA methylation biomarkers, thus has great significance for the implementation of precision medicine for breast cancer.

In healthy individuals, 70%–80% of CpG sequences are in a methylated state, which is very important for maintaining body functions (Sulewska et al., 2007). In tumor cells, specific genes experience high methylation of CpG islands, termed CpG island methylator phenotype (CpG island methylator phenotype, CIMP). Different tumors have different detection sites for CIMP, but the biological mechanisms and pathogenesis of most CIMPs have not been clearly studied. Therefore, there are no unified identification methods and standards. On the other hand, the activity differences of epigenetic markers between different tumor samples are enormous, and most do not follow a normal distribution, limiting the application of many existing statistical methods and mathematical modeling. Thus, it is necessary to develop new algorithms for tumor DNA methylation signature identification and modeling.

In this study, we performed nonparametric permutation test to screen aberrant DNA methylation at the tissue level in the TCGA-BRCA dataset. Then, we conducted non-negative matrix factorization (NMF) analyses to identify DNA methylation signatures associated with BRCA progression. Subsequently, the blood cf-DNA methylation levels of probes in these aberrant DNA methylation signatures were assessed in BRCA patients, and their immunotherapy response with remarkable predictive efficacy and high sensitivity were estimated. Our study provides new insights in epigenetic modification and molecular mechanism in BRCA development.

## 2 Materials and methods

### 2.1 Data sources

Paired DNA Methylation chip data (Illumina Infinium 450 K methylation microarray data) and gene expression of BRCA patients were downloaded from TCGA database, comprising 447 tumor and 52 paired normal samples. The corresponding clinical data including cancer stages, survival time, and tumor purity, was also downloaded. Another DNA methylation data based on peripheral blood were obtained from GEO database under accession number (GSE214344).

### 2.2 Identification of differential methylation sites (DMPs)

On the basis of our previously constructed method for identifying tumor-specific methylation markers (Qin et al., 2024), we screened differential methylation sites (DMPs). Firstly, we performed probe quality control by: 1) removing probes with more than 90% missing values in samples, 2) excluding sites located on the X and Y chromosomes, and 3) eliminating non-probe sites. Ultimately, we obtained 383,561 sites. Subsequently, we selected tumor tissues with paired samples and with the tumor purity greater than 0.6 (estimated using the ESTIMATE method). This resulted in a final cohort of 57 BRCA patients with paired tumor and normal samples. Finally, we employed a nonparametric permutation test to compare the paired samples in this cohort. The model is as follows:

$$\beta_{\text{ij}}=\beta_{0}+\beta_{1}\times {\text{TY}}_{i}+{\text{TO}}_{i}+\varepsilon_{\text{ij}}$$

$\beta_{\text{ij}}$ is the methylation level (0,1)of the $j_{\text{th}}$ probe of the $i_{\text{th}}$ sample, ${\text{TY}}_{i}$ is the tissue type of the $i_{\text{th}}$ sample (cancer or paracancerous tissue), $\beta_{0}$ is the intercept, $\beta_{1}$ is the coefficient related to tissue type, $\varepsilon_{\text{ij}}$ is Gaussian error.

According to the aforementioned model, we performed 1,000 permutations for each methylation site to obtain the corresponding *p*-value for the methylation level between cancer tissues and adjacent normal tissues. Probes with a *p*-value less than 0.001 were defined as BRCA associated DMPs. Then, we calculated the difference value of methylation level (∆β = β_cancer - β_normal) for each sample in the cohort of 57 paired samples. Probes with ∆β > 0 in 80% of the samples were defined as putative hypermethylated positions (Hyper-DMPs), while probes with ∆β < 0 in 80% of the samples were defined as putative hypomethylated positions (Hypo-DMPs). This resulted in a total of 51,736 putative Hyper-DMPs and 27,405 putative Hypo-DMPs.

To further determine the methylation level of each probe, we calculated the median ∆β value and ( $\left.\text{median}∆\beta -∆\beta \right)$ value for the 57 samples. Moreover, to better control unspecific variation of the ∆β values, we set up a second-step threshold. Detailly, based on the distribution of median−∆β values, we selected the top 5% of the 51,736 Hyper-DMPs, threshold of 0.4124, as the cutoff for identifying final Hyper-DMPs, and the bottom 5% of Hypo-DMPs (*n* = 27,405), the threshold of −0.4125, to identify final Hypo-DMPs (Supplementary Figure S1).

### 2.3 DNA methylation signature screening

We performed methylation signature profiling in BRCA patients according to previously published methods (Qin et al., 2024). Firstly, we selected a BRCA population from the TCGA database with tumor tissue purity greater than 0.6 (N = 447). Then, we applied non-negative matrix factorization (NMF) (Lee and Seung, 2000) on the Hyper-DMPs and Hypo-DMPs from the 447 samples. We used two different algorithms, nsNMF (using Kullback-Leibler divergence for multiplicative updates) and Lee (based on Euclidean distance), to perform matrix factorization. Each matrix was iterated 100 times. NMF is an unsupervised learning algorithm that decomposes a non-negative matrix into two matrices, show as following.

$$M_{p\times n}=E_{p\times k}\times S_{k\times n}$$

M is a p×n matrix of methylation, where $p=2,587\;\left(\text{Hyper}-\text{DMPs}\right);\;p=1,370\left(\text{Hypo}-\text{DMPs}\right)$ , and n=447 . S is a matrix of k methylation signatures for n tumor samples, and matrix E is a weight matrix of p probes for k methylation signature.

The optimal number of labels was selected based on the cophenetic coefficient and average silhouette widths. Finally, we obtained three high methylation labels (Hyper-S1, Hyper-S2, and Hyper-S3) and five low methylation labels (Hypo-S1, Hypo-S2, Hypo-S3, Hypo-S4, and Hypo-S5). NMF classified the probes based on their contribution to the labels’ weights, including: Hyper-DMPs:890 probes in Hyper-S1, 855 in Hyper-S2, 842 in Hyper-S3; Hypo-DMPs: 335 in Hypo-S1, 287 in Hypo-S2, 316 in Hypo-S3, 188 in Hypo-S4, and 244 in Hypo-S5.

### 2.4 Immunotherapy response assessment

Several evaluation indices were used to assess the classification performance of methylation probes for immunotherapy response, including sensitivity, specificity, precision, and accuracy. Sensitivity refers to the ability of the probes to identify positive events (in this case, immune therapy response). Specificity refers to the ability to identify negative events (non-response). Precision considers the false positive rate, defined as the number of incorrectly predicted responses divided by the total number of predicted responses. Accuracy measures the proportion of true results (both true positives and true negatives) among the total number of cases examined. Four

$$\text{Sensitivity}=\frac{\text{TP}}{\text{TP}+\text{FN}}$$

$$\text{Specificity}=\frac{\text{TN}}{\text{TN}+\text{FP}}$$

$$\text{Precision}=\frac{\text{TP}}{\text{TP}+\text{FP}}$$

$$\text{Accuracy}=\frac{\text{TP}+\text{TN}}{\text{TP}+\text{TN}+\text{FP}+\text{FN}}$$

TP: True positive rate; TN: True negative rate; FP: False positive rate; FN: False negative rate.

Furthermore, the predictive abilities of the DNA methylation probes were estimated using under the curve (ROC AUC). The calculation formula of the AUC is: $AUC=\int_{0}^{1}TRP\left(FPR^{-1}\left(t\right)\right)dt$

In this formula, TRP is the true positive rate (sensitivity); FPR-1(t) is the inverse function of the false positive rate (1-specificity), representing the false positive rate corresponding to the true positive rate t; the integral range is from 0 to 1, covering the entire range of the ROC curve.s.

### 2.5 Relationship between overall survival, stages and DNA methylation signature activities

To evaluate the correlation between DMP signatures and clinical factors, we compared signature activity differences between primary (stages I and II) and advanced tumors (stages III and IV) using the Wilcoxon signed rank-sum test. Patients were assigned to high- and low-signature groups based on the median signature activity as a cutoff. Differences in overall survival between high- and low-signature groups were evaluated using the Cox proportional hazards regression model via the “survival” R package and visualized with the “ggsurvfit” package.

### 2.6 Validation of methylation signatures’ predictive power for immunotherapy response

Programmed death-ligand 1 (PD-L1) regulates T cell exhaustion by binding programmed death-1 (PD-1) on T cells. Cancer cells with high amounts of PD-L1 can turn T cells off and inhibit T cells attacks, against which immunotherapy medicines (immune checkpoint inhibitors, ICIs) may be effective. PD-L1 is a widely used predictive biomarker for immunotherapy response (Teng et al., 2018). Higher expression of PD-L1 typically correlate with greater therapeutic benefit from ICIs (Jiang et al., 2019). To validate the immunotherapy predictive power of identified methylation signatures, we assessed the relationship between signature activities and PD-L1 gene expression (Transcript per million, TPM) based on paired BRCA DNA methylation data and RNA transcriptome dataset from the TCGA database using cor.test.

### 2.7 Functional enrichment analysis

Genes containing DMPs in the methylation signatures were extracted. Gene ontology (GO) and KEGG pathway enrichment analysis were performed on these genes using Enrichr (Chen et al., 2013).

### 2.8 Statistical analysis

Statistical analysis and plotting were performed using R software. All statistical significances were set as *p* or adjusted *p*-value <0.05.

## 3 Results

### 3.1 DMPs in breast cancer

Based on the 450K DNA methylation chip data from TCGA-BRCA, a total of 92 paired tumor samples and 383,561 methylation sites were selected after quality control. The permutation test method screened 148,494 (38.71%) differential methylation sites (DMPs). According to the 5% cutoff values of Hyper-DMPs (0.4124, *n* = 2,587) and Hypo-DMPs (−0.4125, *n* = 1,370) (Supplementary Figure S1), we finally obtained 2,587 Hyper-DMPs and 1,370 Hypo-DMPs. The amount of Hyper-DMPs is almost twice the number of Hypo-DMPs.

### 3.2 DNA methylation signatures in breast cancer

**FIGURE 1:**
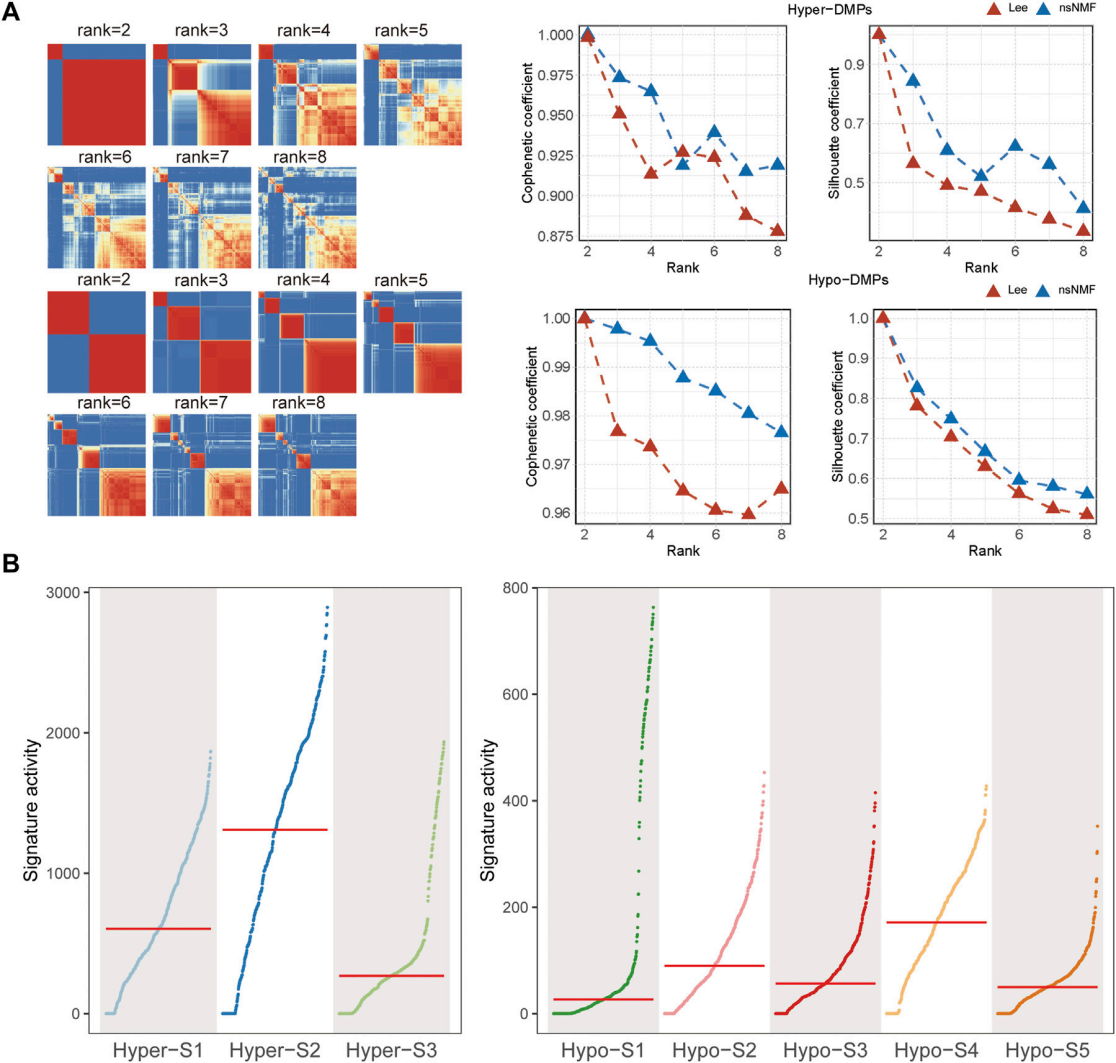
The **(A)** cophenetic, silhouette coefficients and **(B)** activity of DNA methylation signatures based on the (non-negative matrix factorization) NMF method.

Given the non-negativity of DNA methylation levels, the NMF method was subsequently used to extract DNA methylation signatures. Based on the cophenetic coefficient and average silhouette widths of NMF method (Figure 1A), we obtained three high methylation signatures (Hyper-S1, Hyper-S2, and Hyper-S3) and five low methylation signatures (Hypo-S1, Hypo-S2, Hypo-S3, Hypo-S4, and Hypo-S5) from 447 BRCA patients (Figure 1B).

### 3.3 Genomic distribution of DNA methylation signatures associated DMPs in breast cancer

**FIGURE 2:**
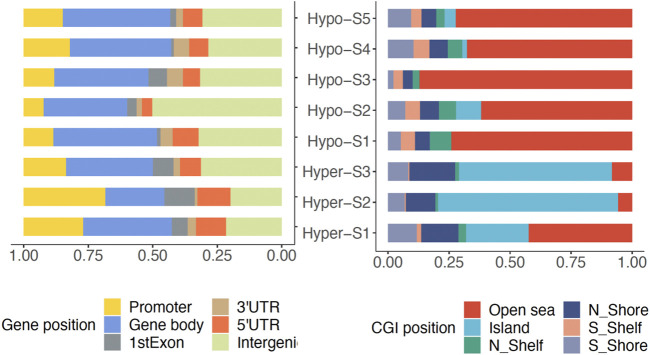
The genomic location of DMPs associated with DNA methylation signatures in gene and CpG island (CGI).

To analyze the genomic characteristics of methylation signatures, we analyzed the genomic distribution features of DMPs associated with each methylation signature. We found that those DMPs associated with high methylation signatures (Hyper-) were mostly located in promoter regions, while DMPs associated with low methylation labels (Hypo-) were mainly located in gene body regions (Figure 2). In terms of CGI position, the Hyper-DMPs were mostly located in CpG islands, the Hypo-DMPs were mostly located in Open Sea areas (Figure 2).

### 3.4 Relationship between DNA methylation signatures and breast cancer stages or overall survival

**FIGURE 3:**
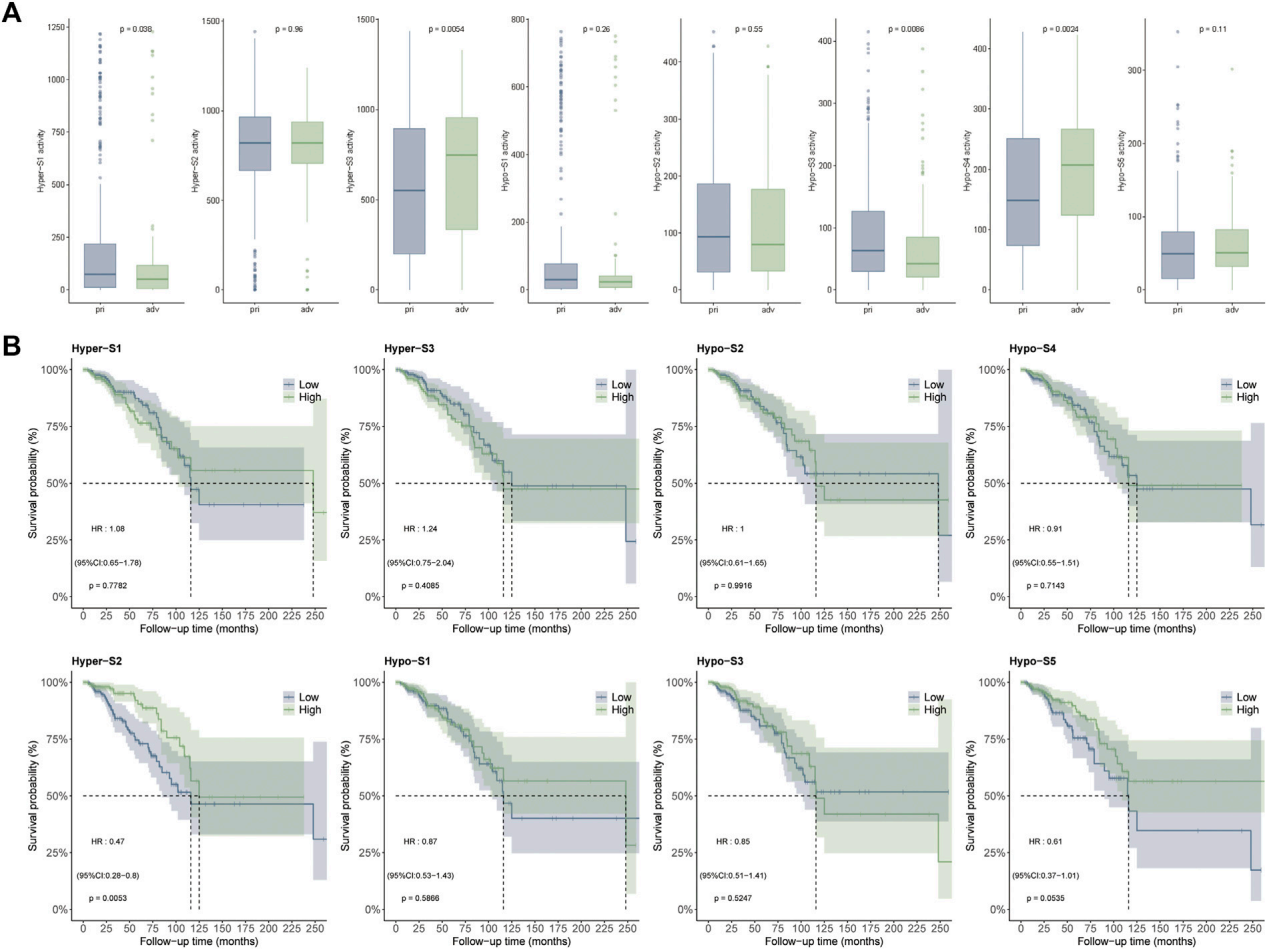
Correlation between DNA methylation signatures and **(A)** tumor stages including primary (stage I-II) and advanced (stage III-IV) stages, and **(B)** survival times.

Since the DNA methylation signatures were tumor-related, whether they were related to tumor progression remained to be answered. To clarify this question, we performed correlation analysis between DNA methylation signature and tumor stage including primary (stage I-II) and advanced (stage III-IV) stages. The Wilcoxon’s rank sum text showed that four out of eight (50%) DNA methylation signatures exhibited markedly differences between the two stages. Detailly, Hyper-S3 and Hypo-S4 were extremely higher in advanced stages. However, Hyper-S1 and Hypo-S3 were significantly upregulated in the primary stages of tumors. The distinct pattern between these four signatures and tumor progression indicates that these four signatures might act vital roles in tumor development (Figure 3A).

DNA methylation alterations can influence tumor development and patient outcomes. To assess the impact of the methylation signatures on BRCA patient’s survival, we compared overall survival times between groups stratified by high and low signature activity levels. Our results showed that patients with low activities of Hyper-S2 signature exhibited significantly longer survival times than those with high Hyper-S2 activities (Figure 3B), indicating its prognostic value. Unlike the tumor stage, only a small proportion (1/8) of signatures affected overall survival. This implies DNA methylation may have a greater impact on tumor progression than direct effects on the survival of BRCA. We therefore focused subsequent analyses on the signatures associated with tumor stage, as these are likely to provide further mechanistic insights into BRCA pathogenesis.

### 3.5 The DMPs from signature Hypo-S4 DNA exhibit significantly lower methylation levels in the blood of BRCA patients than the healthy

**FIGURE 4:**
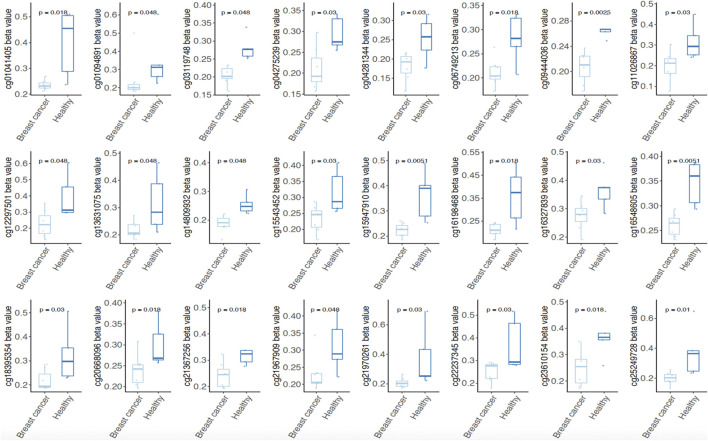
Both of 24 significantly different probes in Hypo-S4 signature showing significantly lower methylation level in BRCA patients than in healthy individuals.

To clarify whether the probes of methylation signatures in tumor tissues are consistent with the methylation level in circulating cell-free DNA of BRCA patients, we further mined the four methylation signatures (Hyper-S1 = 890, Hyper-S3 = 842; Hypo-S3 = 316, Hypo-S4 = 188) related to tumor stage in the methylation data of peripheral blood from five healthy individuals and eight breast cancer patients. We analyzed the probes with abnormal methylation in BRCR peripheral blood within these four methylation labels, and found that 510 probes were associated with tumor blood abnormal methylation (*p* < 0.05), including 137 sites in Hyper-S1, 307 in Hyper-S3, 42 in Hypo-S3, and 24 in Hypo-S4. Notably, we found that the 24 significantly different probes in Hypo-S4 all showed significantly lower methylation in BRCA patients than in healthy individuals (Figure 4), suggesting that these probes could serve as molecular markers for early breast cancer diagnosis.

### 3.6 Relationship between probes with abnormal methylation in DNA methylation signatures and responses to immune therapy, and potential molecular functions

**FIGURE 5:**
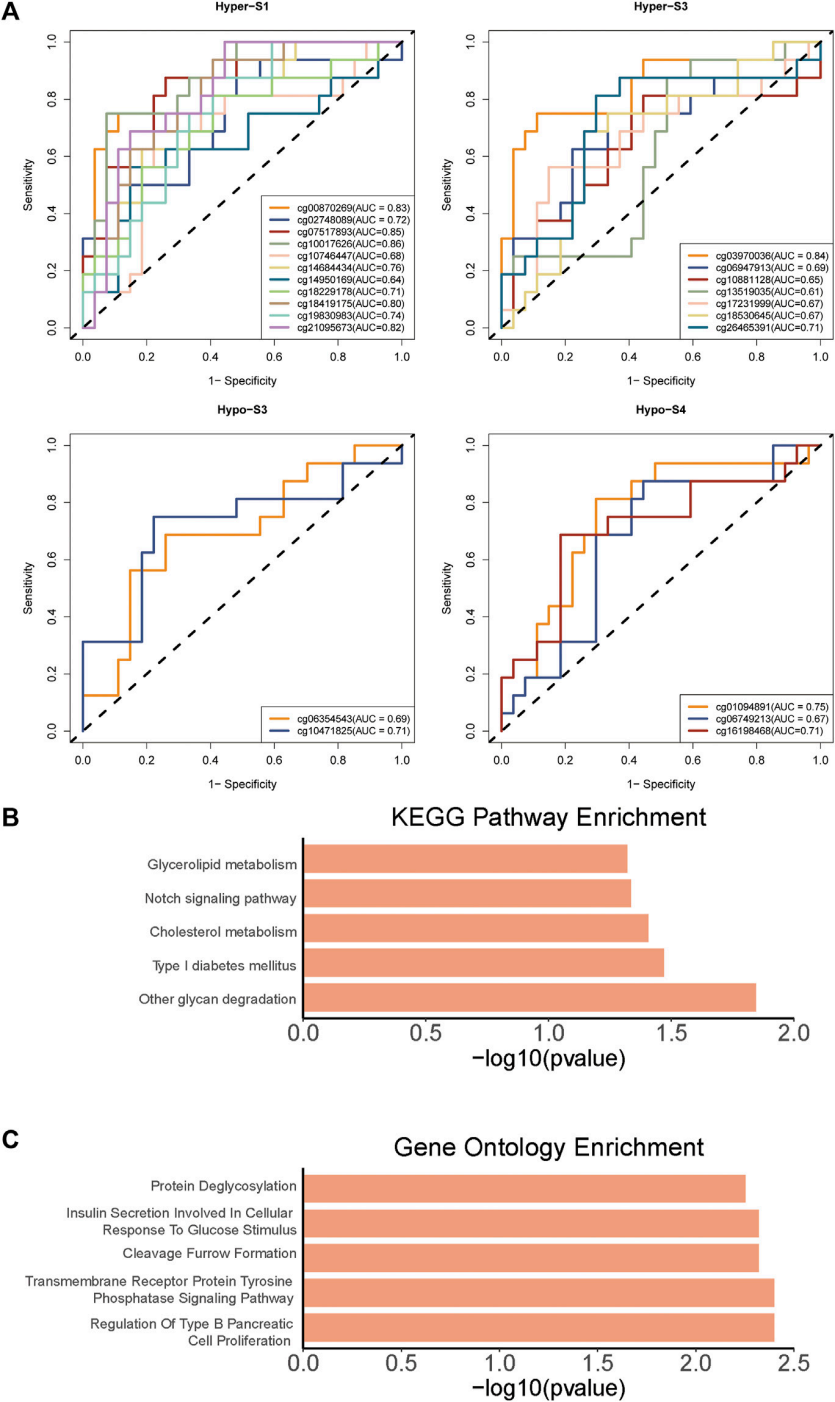
The **(A)** AUC curves of 23 probes associated with patients’ immune therapy response from four tumor stage related DNA methylation signatures, and **(B)** KEGG pathways and **(C)** Gene Ontology enrichments of 23 probes associated genes.

To further validate the relationship between these 510 probes with abnormal methylation and immune therapy efficacy, we performed AUC analysis. Our results showed that 23 probes were associated with patients’ immune therapy response, including 11 probes in Hyper-S1, 7 in Hyper-S3, 2 in Hypo-S3, and 3 in Hypo-S4 (Figure 5A). Their highest predictive power for immune therapy response was 86% (AUC, Table 1). Among them, cg00870269 had a sensitivity of 93%, precision of 86%, accuracy of 74%, and specificity of 66%. cg10017626 had a sensitivity of 75%, precision of 86%, accuracy of 86%, and specificity up to 92%. KEGG pathway enrichment analysis revealed that genes associated with these 23 probes are mainly involved in glycan degradation, Type I diabetes mellitus, cholesterol metabolism, notch signaling pathway, and glycerolipid metabolism pathways (Figure 5B, *p* < 0.05). GO biological process enrichment showed that the immunotherapy-related methylated genes are primarily enriched in processes including regulation of Type B pancreatic cell proliferation, transmembrane receptor protein tyrosine phosphatase signaling pathway, cleavage furrow formation, insulin secretion involved in cellular response to glucose stimulus, protein deglycosylation, and very-low-density lipoprotein particle remodeling (top five, Figure 5C).

**TABLE 1:** The 23 probe sites associated with patient immune therapy response and their classification performance in immune therapy response.

DNA methylation signature	Probe site	AUC value	Specificity	Sensitivity	Accuracy	Precision
Hyper-S1	cg00870269	0.83	0.67	0.94	0.74	0.86
Hyper-S1	cg02748089	0.73	0.52	0.88	0.74	0.75
Hyper-S1	cg07517893	0.85	0.74	0.88	0.65	0.88
Hyper-S1	cg10017626	0.86	0.93	0.75	0.86	0.86
Hyper-S1	cg10746447	0.68	0.78	0.69	0.74	0.81
Hyper-S1	cg14684434	0.77	0.59	0.88	0.70	0.79
Hyper-S1	cg14950169	0.65	0.85	0.56	0.70	0.77
Hyper-S1	cg18229178	0.71	0.59	0.81	0.67	0.84
Hyper-S1	cg18419175	0.81	0.59	0.94	0.70	0.94
Hyper-S1	cg19830983	0.75	0.59	0.88	0.70	0.82
Hyper-S1	cg21095673	0.82	0.56	1.00	0.77	0.81
Hyper-S3	cg03970036	0.84	0.89	0.75	0.70	0.94
Hyper-S3	cg06947913	0.69	0.67	0.75	0.70	0.82
Hyper-S3	cg10881128	0.65	0.56	0.81	0.65	0.83
Hyper-S3	cg13519035	0.61	0.48	0.88	0.60	0.86
Hyper-S3	cg17231999	0.67	0.85	0.56	0.26	0.31
Hyper-S3	cg18530645	0.67	0.74	0.69	0.67	0.81
Hyper-S3	cg26465391	0.72	0.70	0.81	0.30	0.43
Hypo-S3	cg06354543	0.70	0.74	0.69	0.28	0.39
Hypo-S3	cg10471825	0.72	0.78	0.75	0.23	0.33
Hypo-S4	cg01094891	0.75	0.70	0.81	0.30	0.44
Hypo-S4	cg06749213	0.67	0.56	0.88	0.30	0.42
Hypo-S4	cg16198468	0.71	0.81	0.69	0.77	0.81

**FIGURE 6:**
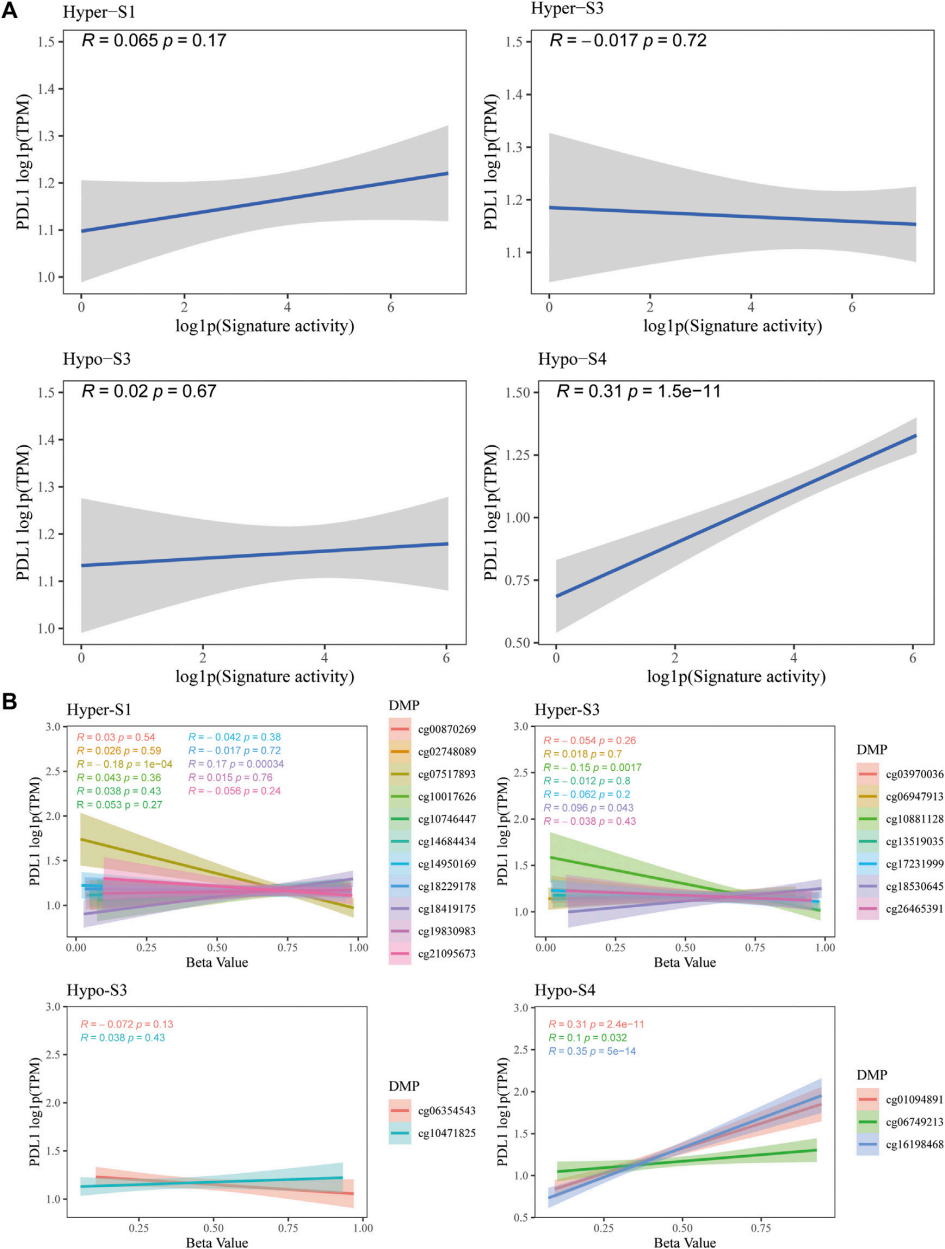
The correlation between PD-L1 gene expression and **(A)** four stage-associated signatures’ activities or **(B)** methylation levels of 23 immunotherapy-response related probes based on the TCGA-BRCA dataset.

The PD-L1 is a biomarker used to assess the effectiveness of ICIs. Therefore, to validate the predictive utility of these signatures and their 23 probes for immunotherapy efficacy, we evaluated the correlation between PD-L1 gene expression and methylation levels from the TCGA-BRCA dataset. Notably, results showed that the activity of the Hypo-S4 signature was positively correlated with PD-L1 expression (Figure 6A). Consistently, three probes in Hypo-S4 associated with immunotherapy response were markedly and positively correlated with PD-L1 gene expression (Figure 6B). While activities of the other three stage-related signatures showed no significant relationship with PD-L1 expression overall (Figure 6A), several individual probes from their corresponding signatures did demonstrate marked correlations, including cg07517893 and cg18419175 from the Hyper-S1 and cg10881128 and cg18530645 from the Hyper-S3 (Figure 6B).

## 4 Discussion

The occurrence and progression of tumors is accompanied by a series of reconstruction processes of the genome and epigenome, and the randomness and complexity of these changes are also the main reasons for the high heterogeneity of tumor cell phenotypes (Ting et al., 2006; Grady and Carethers, 2008). Precision oncology precisely targets specific driving factors in the aforementioned reconstruction processes. Like other tumors, methods for early BRCA diagnosis and screening currently on the market are still limited, especially non-invasive liquid biopsy methods which are extremely scarce.

DNA methylation modification is one of the tumor epigenetic modifications, which is more complex than gene mutations (Kanwal and Gupta, 2012). A prominent feature of tumor genomes is the high methylation of CpG islands in promoters and overall genomic hypomethylation, leading to genome instability and changes in the gene expression profile (Kaneda et al., 2004). In tumor, CpG island methylator phenotype (CIMP) exhibited a specifically high level of methylation. However, due to the heterogeneous and non-normal distribution of epigenetic markers across different tumor types, existing statistical methods and mathematical models have limitations in analyzing DNA methylation patterns. The aberrant and varied methylation profiles between cancer types challenge conventional approaches. Therefore, there is a need for new computational techniques to better capture the distinct methylation signatures and their clinical implications in personalized oncology. In this study, we used permutation tests to screen for differentially methylated loci, and NMF to simplify BRCA’s methylation labels (Qin et al., 2024), which provides prognostic DNA methylation signatures for BRCA. One to be noted, presently, there is no standard identification method for methylation signatures, conclusions deduced from comparisons of different methylation signatures should consider the effects of distinct sizes of differential DMPs.

In total, 148,494 (38.71%) differential methylation sites (DMPs) between BRCA and normal controls were screened. The much higher number of hyper-methylated DMPs than the hypomethylated DPMs is consistent with previous studies (Sun et al., 2021; Campagna et al., 2022; Janssens et al., 2023). Based on these sites, eight methylation signatures were identified. DMPs from high methylation signatures were mostly located in promoter regions or CpG islands, and low methylation DMPs were mainly located in gene body regions or Open Sea areas. These proofs suggest that these signatures are biologically meaningful and can represent differences in methylation patterns between tumor and normal samples.

Furthermore, among the eight DNA methylation signatures, Hyper-S3 and Hypo-S4 were associated with more advanced tumor stages, while Hyper-S1 and Hypo-S3 were upregulated at earlier tumor stages in tissue. This indicates their potential regulatory roles in cancer development. In contrast, only one signature showed an impact on overall survival. The greater number of stage-correlated DNA methylation suggested that they may have a greater impact on tumor progression than direct effects on the survival of BRCA. Subsequent analysis focused mainly on four signatures associated with tumor stages. The 137 sites in Hyper-S1, 307 in Hyper-S3, 42 in Hypo-S3, and 24 in Hypo-S4 exhibited abnormal methylation in blood, suggesting their potential clinical predictive value. Notably, the 24 probes in Hypo-S4 were significantly and commonly hypomethylated in both tumor tissues and blood of BRCA patients, its demonstrating its potential as an molecular marker for early breast cancer diagnosis.

Studies have validated that genome methylation signatures are significantly associated with cancer immunotherapy response (Xu et al., 2021; Qin et al., 2024; Ressler et al., 2024). Consistently, we found that 23 abnormally methylated probes in BRCA blood and tissues were associated with patients’ immune therapy response. ICIs efficacy varies greatly between tumor types and across individual patients due to heterogeneity (Teng et al., 2018). PD-L1 expression is a widely used predictive biomarker of response to cancer ICIs immunotherapy (Davis and Patel, 2019; Doroshow et al., 2021). Significantly positive correlations between PD-L1 gene expression and Hypo-S4 and their three probes (cg01094891, cg06749213, and cg16198468) further validated their predictive values of ICI benefits. Besides the hypomethylated levels of Hypo-S4 in both tissue and blood levels, these proofs suggested the Hypo-S4 signature and its three methylation probes can serve as biomarkers for diagnosis BRCA and its efficacy of immune therapy, providing a theoretical basis for precision treatment.

Cellular glycosylation is a highly organized process involving the addition and modification of glycan residues on proteins and lipids, regulated by glycosyltransferases and glycosidases (Paulson, et al., 1989). Changes in glycosylation pathway expression or activity have been implicated in critical aspects of tumor development and metastasis (Demetriou et al., 1995; Couldrey and Green, 2000). For example, glycan-related alterations can occur early in the carcinogenesis and correlate with BRCA prognosis (Potapenko et al., 2015). The glycosylation -related gene MGAT3 expression is epigenetically regulated by DNA hypomethylation, leading to synthesis of the unique type N-glycans on ovarian cancer cell membrane proteins (Anugraham et al., 2014). In this study, the functional enriched “glycan degradation” KEGG pathway and “protein deglycosylation” GO term commonly revealed immunotherapy response-related probes may impact tumor stages or immunotherapy efficacy by regulating glycan-related pathways. However, further validation in independent patient cohorts of larger size is needed to fully validate the clinical utility of these findings. Additionally, experimental investigation of the underlying molecular mechanisms, using *in vitro* and *in vivo* models, will help elucidate how these differentially methylated positions functionally regulate tumor biology and immunotherapy response. Addressing these limitations in future studies could strengthen the clinical applicability and biological insight provided by this work. And like other epigenetic biomarkers, methylation biomarkers are of great significance for prognosis and diagnosis of cancer (Hao et al., 2017), including BRCA. This study identified DNA methylation signatures and immune therapy-associated DNA methylation sites provide potential diagnostic and therapeutic targets for BLCA treatment.

## 5 Conclusion

Four DNA methylation signatures (Hyper-S3, Hypo-S4, Hyper-S1 and Hypo-S3) of BRCA were identified and found to be associated with tumor progression. A total of 23 abnormally methylated probes identified from these four signatures were associated with patients’ response to immune therapy and may serve as diagnostic markers for predicting the efficacy of immune therapy treatments. Notably, the signature Hypo-S4 and its three hypomethylated sites (cg01094891, cg06749213, and cg16198468) demonstrated potential as good molecular markers for BRCA diagnosis and response to immunotherapy. This study provides new insights into potential molecular markers and targets, offering novel methods and theoretical basis for advancing precision medicine approaches for BRCA.

## Data Availability

Publicly available datasets were analyzed in this study. This data can be found here: TCGA and GEO databases.
