# Supplementary material for: DNA methylation signatures provide novel diagnostic biomarkers and predict responses of immune therapy for breast cancer
Source: Front Genet. 2024 Jun 6;15:1403907. doi: 10.3389/fgene.2024.1403907 (PMC11190699; doi:10.3389/fgene.2024.1403907)
Supplement: Supplementary file 1 [file Image1.pdf]

Supplementary Material

1 Supplementary Figures

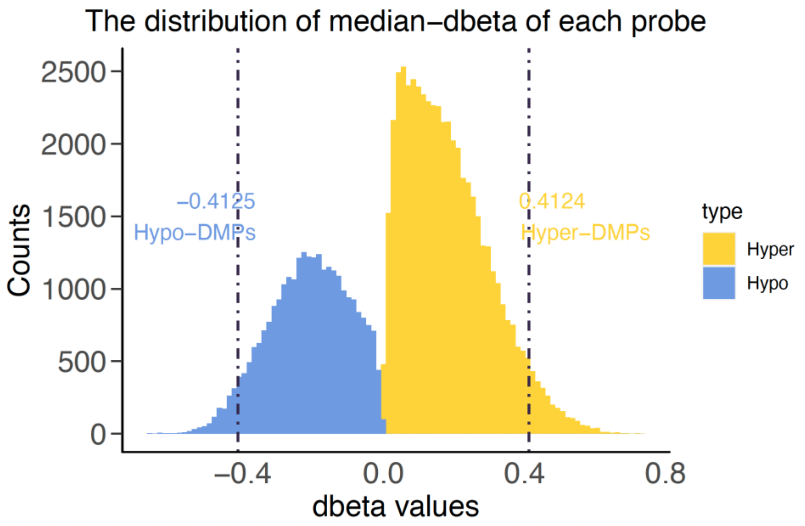

Supplementary Figure 1. Distribution of median − Δβ values of DNA methylation level.
